# Enhanced glycolysis and HIF-1α activation in adipose tissue macrophages sustains local and systemic interleukin-1β production in obesity

**DOI:** 10.1038/s41598-020-62272-9

**Published:** 2020-03-27

**Authors:** Monika Sharma, Ludovic Boytard, Tarik Hadi, Graeme Koelwyn, Russell Simon, Mireille Ouimet, Lena Seifert, Westley Spiro, Bo Yan, Susan Hutchison, Edward A. Fisher, Ravichandran Ramasamy, Bhama Ramkhelawon, Kathryn J. Moore

**Affiliations:** 1 Department of Medicine, Marc and Ruti Bell Program for Vascular Biology and Disease, The Leon H. Charney Division of Cardiology, New York University Langone Health, New York, NY USA; 2 Department of Surgery, New York University Langone Health, New York, NY USA; 3 Department of Medicine, Division of Endocrinology, New York University Langone Health, New York, NY USA

**Keywords:** Mechanisms of disease, Obesity

## Abstract

During obesity, macrophages infiltrate the visceral adipose tissue and promote inflammation that contributes to type II diabetes. Evidence suggests that the rewiring of cellular metabolism can regulate macrophage function. However, the metabolic programs that characterize adipose tissue macrophages (ATM) in obesity are poorly defined. Here, we demonstrate that ATM from obese mice exhibit metabolic profiles characterized by elevated glycolysis and oxidative phosphorylation, distinct from ATM from lean mice. Increased activation of HIF-1α in ATM of obese visceral adipose tissue resulted in induction of IL-1β and genes in the glycolytic pathway. Using a hypoxia-tracer, we show that HIF-1α nuclear translocation occurred both in hypoxic and non-hypoxic ATM suggesting that both hypoxic and pseudohypoxic stimuli activate HIF-1α and its target genes in ATM during diet-induced obesity. Exposure of macrophages to the saturated fatty acid palmitate increased glycolysis and HIF-1α expression, which culminated in IL-1β induction thereby simulating pseudohypoxia. Using mice with macrophage-specific targeted deletion of HIF-1α, we demonstrate the critical role of HIF-1α-derived from macrophages in regulating ATM accumulation, and local and systemic IL-1β production, but not in modulating systemic metabolic responses. Collectively, our data identify enhanced glycolysis and HIF-1α activation as drivers of low-grade inflammation in obesity.

## Introduction

Obesity and its associated metabolic diseases, insulin resistance and type 2 diabetes, continue to increase in the United States and worldwide^1^, and fully effective therapies to combat this growing epidemic are lacking. Obesity gives rise to a state of chronic inflammation, which is causally linked to insulin resistance (IR) and type 2 diabetes^2–4^. In obese mice and humans, there is a striking accrual of macrophages in visceral adipose tissue ^5,6^, and these immune cells are key contributors to obesity-induced insulin resistance^2,7^. Whereas adipose tissue macrophages (ATM) comprise approximately 10–15% of stromal cells in the lean adipose tissue, during high fat diet feeding the recruitment of monocytes into visceral adipose depots can drive macrophage accumulation up to 50% of all cells in obese adipose tissue^7^. A number of factors, including adipocyte hypertrophy, increased extracellular lipid concentrations and oxidative stress are thought to drive expression of chemoattractant factors that recruit monocytes in the expanding adipose tissue^2,7^. In this metabolically disturbed environment, the monocyte-derived macrophages take on an inflammatory phenotype that is distinct from those of macrophages residing in lean adipose tissue^8,9^. These ATMs are a source of inflammatory cytokines, particularly TNFα^10^ and IL-1β^11^, that sustain the local and systemic inflammation that contributes to insulin resistance [reviewed in^2^], yet the mechanisms that drive metabolic inflammation and the pro-inflammatory ATM phenotype, remain poorly understood.

Macrophages can take on a spectrum of activation states to fulfill a broad range of functions needed for host defense, homeostasis, and tissue repair^12^. *In vitro* characterization of macrophage activation programs has led to the description of classically activated (by LPS + interferon-γ M1 macrophages (M[LPS + IFNγ]) and alternatively activated (by IL-4) M2 macrophages (M[IL-4]), which mediate pro- and anti-inflammatory macrophage functions^13^. However, *in vivo*, there is likely an array of macrophage activation states that are dictated by the local tissue microenvironment. Indeed, gene expression profiling of ATM from obese mice and humans has shown that these macrophages have some, but not all, features of pro-inflammatory M[LPS + IFNγ] macrophages^8,14^. Recent studies indicate that rewiring of cellular energy metabolism is a key feature of macrophage activation that directly impacts macrophage phenotype and function^15^. For example, alternatively activated M[IL-4] macrophages exhibit enhanced mitochondrial oxidative phosphorylation (OXPHOS) to fuel their function^16^. By contrast, classically activated M[LPS + IFNγ] macrophages have enhanced glycolysis that provides a rapid source of ATP from glucose to sustain their high secretory and phagocytic functions, and also feeds the pentose phosphate pathway to generate amino acids for protein synthesis, ribose for nucleotides and NADPH for the production of reactive oxygen species (ROS) by NADPH oxidase^17^. Although these metabolic programs were originally thought to merely reflect the cell’s energy substrate utilization, recent studies using chemical or genetic inhibitors of glycolysis and fatty acid oxidation have shown that disrupting these programs can alter macrophage phenotype and inflammatory functions^16–18^. Together, these findings have led to an appreciation that distinct bioenergetics profiles can guide macrophage functional responses in tissues, however the cellular metabolic programs of ATM are largely unexplored.

Hypoxia inducible factor 1 alpha (HIF-1α) is a potent transcriptional regulator of cellular adaptation programs required for growth and survival under hypoxic conditions, particularly the switch to glycolysis as OXPHOS becomes limited under low oxygen concentrations^19^. Studies in obese mouse models and humans have documented the accumulation of HIF-1α in both adipocytes and ATM, and this has been attributed to oxidative stress that develops in the expanding adipose tissue^20,21^. HIF-1α activation has also been shown to occur in LPS-activated macrophages under normoxic conditions (pseudohypoxia), which potentiates glycolytic flux at the expense of OXPHOS, and drives inflammatory gene expression, most notably of the pro-inflammatory cytokine IL-1β^17^. This has been linked to the accumulation of TCA cycle intermediates, such as succinate^15^, which along with ROS can stabilize HIF-1α by inhibiting prolyl hydroxylases (PHDs) that promote its degradation. Whether pseudohypoxia contributes to activation of HIF-1α in metabolically activated ATM, and their inflammatory response, is poorly understood

Here, we hypothesize that in obesity, metabolic dysregulation leads to both hypoxic and pseudohypoxic stimuli that activate HIF-1α in ATM, thereby sustaining systemic low-grade inflammation, a condition that fuels insulin resistance. Characterization of the metabolic phenotype of ATM of lean and obese mice revealed that unlike M[LPS + IFNγ] macrophages, which exhibit elevated glycolysis and impaired mitochondrial OXPHOS, ATM from high fat diet fed mice display increases in both glycolysis and OXPHOS programs compared to ATM from lean mice. Notably, HIF-1α, IL-1β and genes in the glycolysis pathway levels were increased in ATM isolated from white adipose tissue (WAT). Using a hypoxia tracer, we show that HIF-1α accumulates in both hypoxic and non-hypoxic ATM, suggesting that both low oxygen conditions and pseudohypoxia could contribute to HIF-1α driven inflammation *in vivo*. Incubating macrophages with palmitate, a saturated fatty acid released by adipocytes during lipolysis, under normoxic conditions, was sufficient to increase glycolysis and basal OXPHOS, as well as the HIF-1α and IL-1β production. Notably, the increased expression of IL-1β and genes in the glycolysis pathway induced by palmitate was reversed upon incubation with a HIF-1α inhibitor, implicating this transcription factor as a central regulator of the metabolically activated macrophage phenotype. Finally, we show that targeted deletion of HIF-1α, specifically in myeloid cells, reduced ATM accumulation in the WAT of high fat diet fed mice, as well as local and systemic IL-1β production, implicating HIF-1α as a guardian of metabolic stress and inflammation in obesity.

## Results

### Obesity reprograms cellular energy metabolism in adipose tissue macrophages

**Figure 1:**
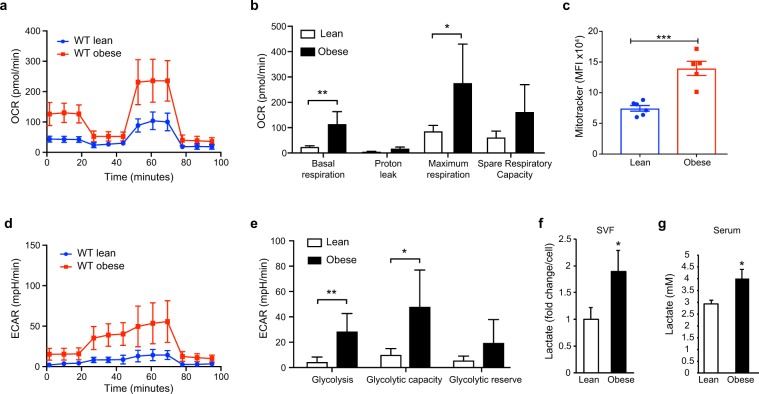
(**a**–**d**) Seahorse analysis of cellular (**a**) oxygen consumption rate (OCR), (**b**) basal respiration, maximum respiration and spare respiratory capacity, (**c**) extracellular acidification rate (ECAR), and (**d**) glycolysis, glycolytic capacity and glycolytic reserve, in ATM from lean and obese wild type (WT) mice. Data are representative of 2 independent experiments from n = 2–3 mice/group. (**e)** Lactate levels in the stromal vascular fraction (SVF) of lean (n = 4) and obese mice (n = 5). (**f)** Serum lactate levels in lean (n = 6) and obese (n = 9) mice. Data are expressed as mean ± s.e.m. **p < 0.01, *p < 0.05.

Recent studies indicate that rewiring of cellular energy metabolism can regulate macrophage phenotype and function, yet little is known about the metabolic programs that characterize ATM in obesity. To investigate this, we fed mice a low fat chow diet or a high fat diet for 20 weeks and isolated ATM from WAT. Flow cytometric characterization showed that 85–95% of these cells expressed the macrophage markers F4/80, MerTK and CD64, while less than 1% expressed the neutrophil marker Ly6G (Supplementary Fig. 1). Using Seahorse extracellular flux analyses we measured the oxygen consumption rate (OCR) and extracellular acidification rate (ECAR) of ATM to determine OXPHOS and glycolysis, respectively. Compared to ATM from lean mice, ATM from obese mice showed an increase in both basal and maximal respiration, indicative of increased OXPHOS (Fig. 1a,b). As this finding may indicate an increase in mitochondrial mass in ATM from obese mice, we stained ATM from lean and obsese mice with mitotracker dye. We observed increased mitotracker staining in ATM isolated from obese compared to lean mice, indicative of elevated mitochondrial content in ATM residing in obese adipose tissue (Fig. 1c). Notably, ATM from obese mice also showed an increase in ECAR and glycolytic capacity compared to ATM from lean mice (Fig. 1d,e). This was consistent with increased levels of lactate, the by-product of glycolysis, in the stromal vascular fraction of WAT (Fig. 1f) and serum (Fig. 1g) of obese mice compared to lean mice. Together, these data suggest that ATM that accumulate in WAT undergo metabolic remodeling to support the biosynthetic and bioenergetic demands of the cell in the expanding adipose tissue.

**Figure 2:**
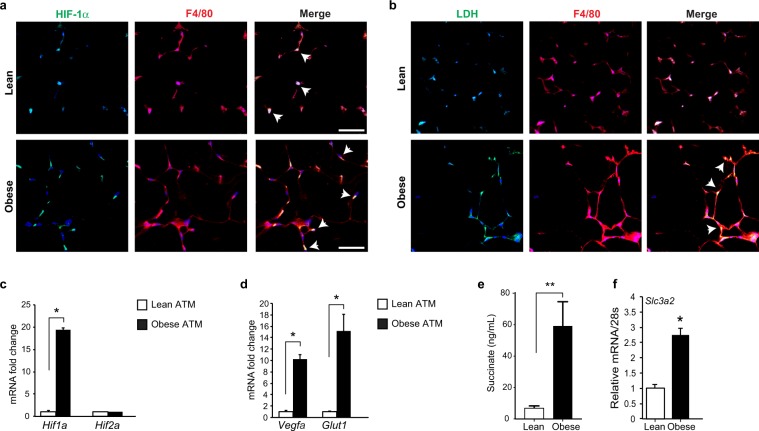
(**a**,**b**) Immunofluorescence staining of (**a**) HIF-1α (green), the macrophage marker F4/80 (red) and DAPI nuclear stain (blue), and (**b**) lactate dehydrogenase (LDH, green), F4/80 (red) and DAPI (blue) in WAT of lean and obese mice. Colocalization is shown in the merged image (arrows). Scale bar = 100 µm. (**c**,**d)** Relative mRNA expression of (**c**) *Hif1a* and *Hif2a*, and the HIF target genes (**d**) *Vegfa* and *Glut1* in ATM isolated from lean and obese mice (n = 3). (**e**) Levels of succinate in ATM of lean and obese mice (n = 6). (**f)** Relative mRNA levels of the glutamate transporter *Slc3a2* in ATM from lean and obese (n = 3). Data is expressed as mean ± s.e.m. *p < 0.05.

Cellular metabolic programs can shape the activation and function of immune cells. For example, a potent transcriptional activator of glycolytic enzymes and inflammatory factors is the transcription factor HIF-1α^19^. Immunofluorescence staining for HIF-1α and the macrophage marker F4/80 showed increased HIF-1α staining that co-localized with DAPI nuclear stain, indicative of active HIF-1α in the nucleus of macrophages in obese adipose tissue compared to lean condition (Fig. 2a, Supplementary Fig. 2). To understand whether the observed increase in glycolysis was associated with activation of HIF-1α protein in ATM from obese mice, we performed immunofluorescence staining for lactate dehydrogenase (LDH), a glycolytic enzyme that catalyzes the final step required for lactate synthesis. In the obese adipose tissue, LDH staining localized to cells in crown-like structures that co-stained for F4/80 (Fig. 2b, Supplementary Fig. 2). Analysis of gene expression in ATM isolated from obese mice show increased expression of HIF-1α target genes, including the glucose transporter *Glut1* and angiogenic factor *Vegfa*, compared to ATM from lean mice (Fig. 2c). Furthermore, ATM from obese mice showed greater expression of *Hif1-*α, but not *Hif2-*α mRNA, than ATM from lean mice (Fig. 2d), consistent with reports that HIF-1α can promote the expression of its own α-subunit through a transactivation loop. In line with HIF-1α activation in ATM from obese adipose tissue, the Kreb’s cycle metabolite succinate, which was previously demonstrated to activate HIF-1α, was increased over 10-fold in ATM of obese compared to lean mice (Fig. 2e). Since succinate can be derived from glutamate, a substrate that can replenish the TCA cycle, we examined expression of the glutamate transporter *Slc3a2*. As shown in Fig. 2f, *Slc3a2* mRNA was increased in ATM from obese compared to lean mice. These data suggested that accumulation of succinate in ATM in obese adipose tissue could also contribute to HIF-1α activation.

### Hypoxia fuels adipose tissue macrophage glycolysis and inflammation *in vivo*

**Figure 3:**
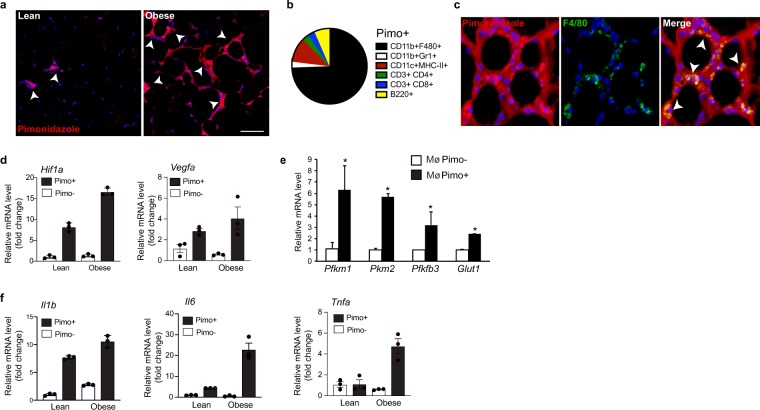
**(a**) Immunohistochemical staining of WAT of pimonidazole-treated lean and obese mice showing pimo adducts (red) and DAPI (blue). Scale bar = 100 µm. Arrows indicate pimo+ cells in crown-like structures. (**b)** Relative distribution of Pimo+ immune cell subpopulations isolated by flow cytometry from WAT of pimonidazole-treated obese mice (n = 3). (**c)** Immunofluorescence staining of the macrophage marker F4/80 (green), the pimonidazole hypoxia probe (red) and DAPI nuclear stain (blue) in WAT of obese mice. Colocalization of pimonidazole with F4/80 is shown in yellow in the merged image (arrows). Scale bar = 100 µm. (**d**–**f)** Relative mRNA levels of (**d**) *Hif1* α and the HIF-1α-dependent gene *Vegfa*, and (**e**) glycolysis-related and (**f**) inflammatory genes in Pimo+ or Pimo^−^ ATM isolated from lean and obese mice (n = 3). Data are expressed as mean ± s.e.m. *p < 0.05.

A number of factors may drive HIF-1α activation and increased glycolysis in ATM of the expanding adipose tissue, including the development of hypoxia and stimuli that can promote a pseudohypoxic state by preventing HIF-1α degradation. To begin to investigate these factors, we injected mice with a chemical hypoxia tracer, pimonidazole, which forms covalent bonds with cellular macromolecules at oxygen levels below 1.3%^22^, in order to visualize and isolate hypoxic cells from WAT of lean and obese mice. Immunofluorescent staining for pimonidazole adducts revealed sparse and scattered staining in WAT of lean mice, whereas pimonidazole adduct accumulation was greatly increased in crown-like structures of the WAT of obese mice (Fig. 3a). To identify the cell types within the obese WAT that localized to the pimonidazole-enriched regions (Pimo+), we isolated the stromal vascular fraction (SVF) from WAT and performed flow cytometry. Notably, the majority of Pimo+ cells (75%) expressed markers of tissue macrophages (CD11b^+^F4/80^+^). We confirmed co-localization of pimonidazole adducts with the macrophage markers F4/80 (Fig. 3c) and CD64 (Supplementary Fig. 3) in crown-like structures of WAT from obese mice by immunofluorescence. To characterize the gene expression profile of these cells, we sorted Pimo^−^ and Pimo^+^ CD11b^+^F4/80^+^ macrophages by fluorescence activated cell sorting (FACS) of live cells from the WAT (gating strategy in Supplementary Fig. 3b) of lean and obese mice and performed qRT-PCR analysis. Pimo^+^ ATM from both lean and obese WAT showed increased mRNA levels of *Hif1a* and HIF-1α-regulated gene *Vegf*, compared to Pimo^−^ ATM, with the highest levels seen in Pimo^+^ ATM from obese WAT (Fig. 3d). Consistent with previous reports that hypoxia increases glucose utilization and glycolysis (18), Pimo^+^ ATM from obese mice showed increased expression of genes associated with glucose uptake (*Glut1*) and glycolysis (*Pfkm1*, *Pkm2*, *Pfkfb3*) compared to Pimo^−^ ATM from the same mice (Fig. 3e). We also noted increased *Pkm2 and Glut1* mRNA levels in Pimo^+^ cells isolated from lean adipose tissue suggesting a dynamic response to hypoxic stress ( Supplementary Fig. 4). Furthermore, Pimo^+^ ATM from WAT of obese mice showed increased expression of inflammatory cytokines, including *Il1b*, which is known to be regulated by HIF-1α in highly glycolytic M1 macrophages (Fig. 3f), as well as *Il6* and *Tnfa* mRNAs. These studies implicate hypoxia-driven glycolysis as a regulator of the inflammatory phenotype of ATM in obese adipose tissue.

### Palmitate upregulates macrophage glycolysis and Hif-1α activation

**Figure 4:**
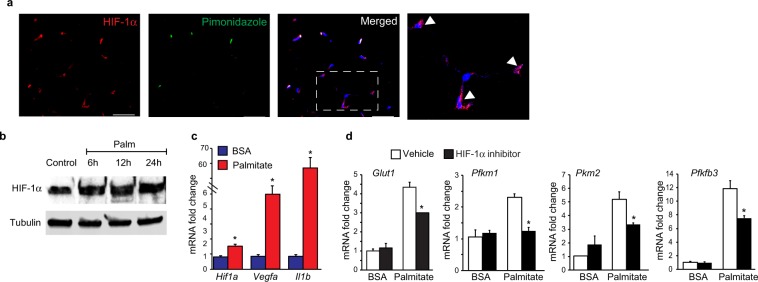
(**a**) Immunofluorescence staining for HIF-1α (red), hypoxia probe pimonidazole (green) and DAPI nuclei (blue) in WAT of obese mice. Boxed region in the merged image shows HIF-1α-positive cells in crown-like structures that are Pimo^−^ (arrows). (**b**) HIF-1α protein levels in BMDM treated with BSA (control) or BSA-conjugated palmitate (Palm) for 6, 12 and 24 hours. Tubulin is shown as an internal loading control. (**c**) Fold change in mRNA expression of *Hif1a, Vegfa* and *Il1b* in BMDM treated with palmitate or BSA. (**d**) Relative mRNA expression in BMDM treated as indicated  in the presence or absence of a HIF-1α inhibitor. Data are representative of 3 independent experiments and are expressed as mean ± s.e.m. *p < 0.05.

**Figure 5:**
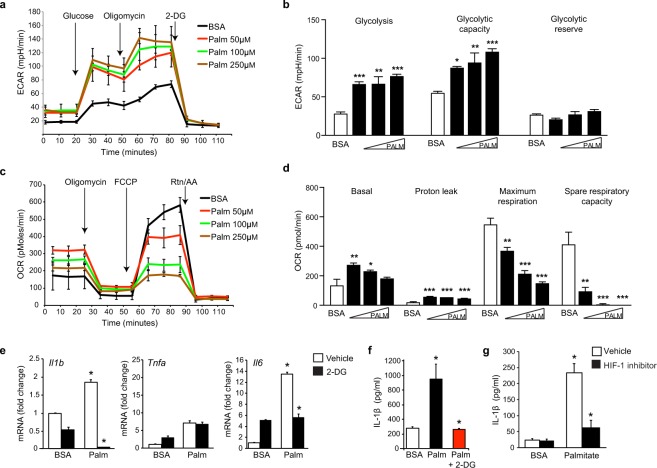
**(a**,**b**) Seahorse analysis of (**a**) extracellular acidification rate (ECAR) and (**b**) glycolysis, glycolytic capacity and glycolytic reserve in BMDM treated with 50, 100 or 250 µM BSA-conjugated palmitate or 250 µM BSA alone. (**c,d)** Seahorse analysis of (**c**) oxygen consumption rate (OCR) and (**d**) basal respiration, maximum respiration and spare respiratory capacity in BMDM treated as indicated. (**e**) Relative mRNA expression of inflammatory cytokines in BMDM treated with 250 µM BSA or BSA-conjugated palmitate in the presence and absence of 2-deoxyglucose (2-DG). Levels of IL-1β protein in the supernatant of BMDM treated with BSA or BSA-palmitate in the presence of absence of (**f**) 2-DG or (**g**) a HIF-1α inhibitor. Data are representative of 3 independent experiments and are expressed as mean ± s.e.m. *p < 0.05.

During our analysis of WAT of obese mice injected with pimonidazole to detect hypoxic cells, we also noted HIF-1α^+^Pimo^−^ cells accumulating in crown-like structures, indicative of HIF-1α activation in the absence of hypoxia. As shown in Fig. 4a, co-staining for HIF-1α and pimonidazole adducts showed only partial co-localizion of HIF-1α and pimonidazole staining, with HIF-1α nuclear staining also localizing to Pimo^−^ cells (boxed region, arrows). We hypothesized that stimuli present in obese adipose tissue may lead to a pseudohypoxic state in ATM that stabilizes HIF-1α protein in the absence of hypoxia. To test this, we treated bone marrow-derived macrophages (BMDMs) with palmitate, a saturated fatty acid released from adipocytes during obesity that has been linked to macrophage activation^8,11^. Treatment of BMDMs with palmitate-BSA caused a time dependent increase in HIF-1α protein (Fig. 4b), compared to BSA treated BMDMs. Consistent with HIF-1α increase, we observed abundant mRNA levels of HIF-1α target genes, including *Vegfa*, *Il1b* (Fig. 4c), *Glut1* and the glycolytic enzymes *Pfk1*, *Pkm2*, and *Pfkfb3* in palmitate-BSA treated BMDMs compared to control BSA treated BMDMs (Fig. 4d). As HIF-1α activation and enhanced glycolysis occurs in LPS-treated macrophages under normoxic conditions, we next performed Seahorse bioenergetics analyses to determine whether palmitate could metabolically reprogram macrophages to drive a similar pseudohypoxic state. Palmitate-BSA dose dependently increased glycolysis and glycolytic capacity in BMDMs compared to BSA alone, even at the lowest dose of palmitate tested (Fig. 5a,b). Furthermore, while palmitate-BSA increased basal respiration in BMDMs as seen in ATM from obese mice, it reduced the maximal respiratory rate and spare respiratory capacity of BMDMs, compared to treatment with BSA alone (Fig. 5c,d). These findings indicate that palmitate treatment of macrophages induces HIF-1α activation and some, but not all, of the metabolic changes seen in ATM from obese adipose tissue.

To determine whether the glycolytic reprogramming of palmitate-treated BMDMs is linked inflammation, we measured the expression of IL-1β, IL-6 and TNF-α in palmitate-BSA or BSA treated BMDMs in the presence and absence of 2-deoxyglucose (2-DG), an inhibitor of glycolysis. Compared to BSA alone, palmitate-BSA increased the expression of *Il1b, Tnfa* and *Il6* mRNA in BMDMs, and the palmitate-induced increases in *Il1b* and *Il6*, but not *Tnfa*, mRNA were abrogated in BMDMs treated with 2-DG, indicating that these responses are dependent on glycolysis (Fig. 5e). Furthermore, we found that palmitate-BSA increased the secretion of IL-1β protein by BMDMs, compared to control BSA treatment, and this was blocked by pretreatment of macrophages with 2-DG or a HIF-α inhibitor (Fig. 5f,g). Together, these results link palmitate-induced glycolysis or HIF-1α activation to the induction of IL-1β in macrophages.

### HIF-1α deletion in myeloid cells induces a phenotypic switch in ATM and tempers WAT inflammation

**Figure 6:**
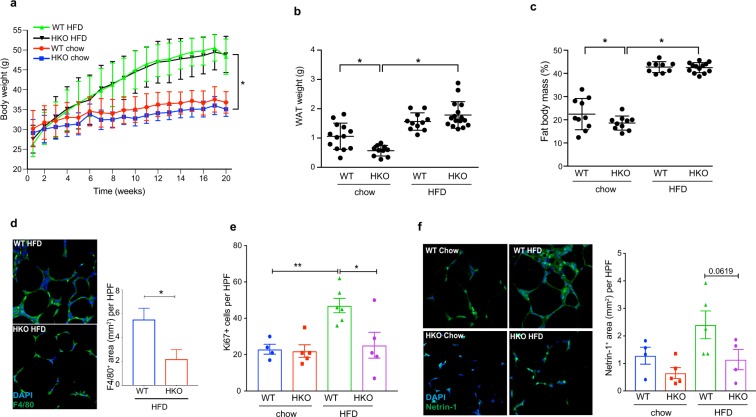
**(a**) Weight of WT and HKO mice fed chow or HFD for 20 weeks. (**b**) WAT weight of WT and HKO mice after 20 weeks of chow and HFD diet. (**c)** Fat mass of WT and HKO mice after 20 weeks of chow or HFD as measured by DEXA scanning. (**a**–**c**) n = 10–12 mice. (**d**) Representative immunofluorescence staining of the macrophage marker F4/80 (green) and DAPI nuclei (blue) in WAT of WT and HKO mice fed HFD for 20 weeks. Quantification of F4/80+ macrophages in WAT shown at right (n = 3). (**e**) Quantification of immunofluorescence staining for the proliferation marker Ki67 in ATM of WT and HKO HFD-fed mice. (**f**) Representative immunofluorescence staining for the macrophage retention cue netrin-1 (green) and DAPI nuclei (blue) in WAT of WT and HKO mice fed HFD for 20 weeks. Quantification shown at right. (**e**,**f**) n = 5 mice/group. Data are expressed as mean ± s.e.m. *p < 0.05.

We next tested the effects of macrophage-specific deletion of HIF-1α on obesity related inflammation and metabolic dysfunction. We fed *Hif1a*^fl/fl^*LysMcre*+ (HKO) and *Hif1a*^fl/fl^*LysMcre*− (WT) mice a low fat chow or high fat diet for 20 weeks. Although no difference in weight gain or fat mass was observed in WT and HKO mice on the HFD, HKO mice fed chow diet had a modest reduction in weight and adipose mass compared to WT mice on chow diet (Fig. 6a,b), which was confirmed by dual-energy X-ray absorptiometry (DEXA) (Fig. 6c). Uncoupling protein 1 (*Ucp1*) mRNA levels in WAT of HKO lean mice was increased compared to WT mice, consistent with an induction of local thermogenesis that could lead to reduced WAT weight in lean HKO mice. Notably, *Ucp1* mRNA expression was decreased in WAT of HFD fed WT and HKO mice compared to chow fed WT and HKO mice, and no difference was observed between the genotypes in the HFD-fed groups (Supplementary Fig. 5a). Brown adipose tissue (BAT) mass was increased upon HFD feeding but no difference was observed between the genotypes studied (Supplementary Fig. 5b). Despite similar adiposity in WT and HKO mice after HFD feeding, we observed a decrease in F4/80^+^ macrophage accumulation in WAT of HKO mice as measured by immunofluorescence staining (Fig. 6d). To investigate the mechanisms contributing to the reduced numbers of F4/80+ cells in HKO HFD-fed mice compared to WT mice, we measured markers of macrophage proliferation and retention. We observed a decrease in the proliferation marker Ki67 in WAT of HFD-fed HKO compared to WT mice (Fig. 6e, Supplementary Fig. 5c) suggesting that macrophage proliferation is reduced in the absence of HIF-1α. Furthermore, we also observed a decrease in the expression of the neuroimmune guidance cue netrin-1, which promotes macrophage survival and tissue retention^23,24^, in HFD-fed HKO compared to WT mice (Fig. 6f). These findings are consistent with previous studies showing that expression of netrin-1 is regulated by HIF-1α in macrophages^25^. These data suggested that targeted deletion of HIF-1α in macrophages reduced the accumulation of these cells in the WAT during obesity.

**Figure 7:**
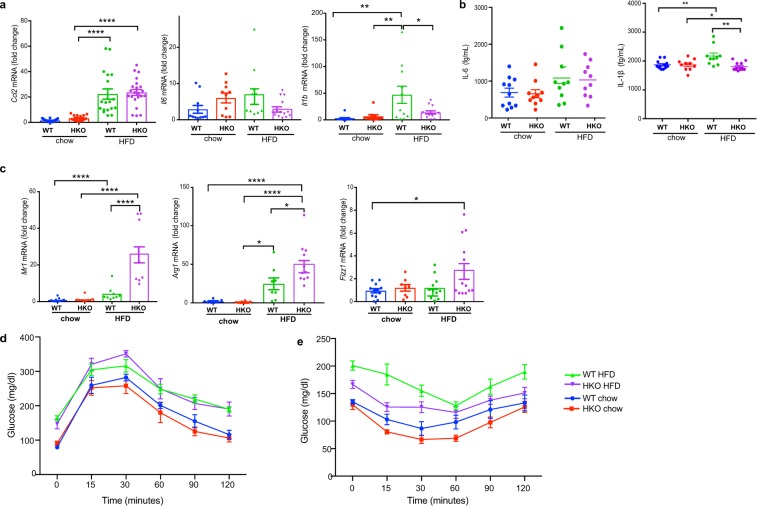
(**a**) Relative mRNA expression of *Ccl2*, *Il6* and *Il1b* in WAT of wild type (WT) and Hif1a^fl/fl^LysMcre+ (HKO) on chow or HFD diet for 20 weeks. (**b**) Plasma levels of IL-6 and IL-1β in WT and HKO mice on chow or HFD for 20 weeks. (**c**) Relative mRNA expression of *Mr1*, *Arg1*, and *Fizz1* in WAT of WT and HKO mice fed chow or HFD for 20 weeks. (**d**) Glucose tolerance test (GTT) and (**e**) insulin tolerance test (ITT) of WT (n = 11) and HKO (n = 10–12) mice fed chow or HFD diet for 20 weeks. Data are expressed as the mean ± s.e.m. ****p < 0.0001, ***p < 0.001, **p < 0.01, *p < 0.05.

Consistent with previous studies we observed an increase in the expression of the macrophage pro-inflammatory mediators, *Ccl2*, *Il6* and *Il1b* in the WAT of WT mice fed HFD compared to chow diet (Fig. 7a). While we observed no difference in *Ccl2* and *Il6* mRNA levels in the WAT of HKO and WT mice fed HFD, *Il1b* mRNA expression was selectively reduced in WAT of HKO mice compared to their WT counterparts. Furthermore, the decreased expression of *Il1b* mRNA in WAT correlated with decreased serum levels of IL-1β in HFD-fed HKO mice compared to HFD-fed WT, as measured by cytometric bead array assay (Fig. 7b). To further understand the effects of HIF-1α deletion on macrophage inflammatory status in WAT during obesity, we measured the expression of a panel of markers that characterize alternatively activated macrophages. The absence of HIF-1α in macrophages enhanced the expression of *Mr1*, *Arg1* and *Fizz1* mRNA in the WAT of HKO mice compared to WT mice fed HFD (Fig. 7c), suggesting increased accumulation of alternatively activated macrophages known to mediate tissue repair. Interestingly, despite these improvements in WAT inflammation and local and systemic IL-1β expression in HFD-fed HKO mice, we observed no significant difference in insulin sensitivity compared to WT mice (Fig. 7d), although there was a trend towards improved glucose tolerance in HFD-fed HKO compared to WT mice (Fig. 7e). Together, these data indicate that HIF-1α drives macrophage expression of IL-1β in the obese adipose tissue, leading to increased concentrations of IL-1β in the plasma. While deletion of HIF-1α reduces local and systemic IL-1β levels, and causes an enrichment of M2 macrophages in the WAT, this is not sufficient to reverse the metabolic dysfunction associated with obesity.

## Discussion

Targeting immunometabolism in chronic inflammatory diseases has emerged as a promising new therapeutic avenue. Recent studies using microbial or cytokine stimuli have begun to establish metabolic signatures for different types of *in vitro* activated macrophages^12,13^. However, tissue microenvironmental cues are important regulators of macrophage metabolism and thus, understanding the metabolic behaviours of tissue macrophages is of fundamental importance in designing new therapeutic approaches. Here we defined the metabolic programs engaged in adipose tissue macrophages in the context of obesity, and demonstrated that this is directly linked to their proinflammatory phenotype via the transcription factor HIF-1α.

Recent studies have shown that macrophages that accumulate in WAT during obesity have an activated phenotype that shares some, but not all features of classically activated LPS-treated macrophages^8,14^. Indeed, while macrophages acutely stimulated with LPS undergo a profound shift in metabolism towards glycolysis at the expense of OXPHOS, we show that ATM isolated from obese mice exhibit upregulation of both glycolysis and OXPHOS as measured by Seahorse Bioenergetic Analysis. These findings are consistent with those of Stienstra and colleagues, who recently published a transcriptomic analysis of ATM that showed upregulation of genes involved in both glycolysis and OXPHOS in ATM of obese compared to lean mice - changes that were distinct from those observed in LPS-stimulated macrophages^26^. Interestingly, this metabolic rewiring in response to HFD-feeding was tissue specific, as peritoneal macrophages from obese mice did not exhibit similar transcriptomic changes. Furthermore, consistent with the metabolic phenotype that we measured in ATM isolated directly from obese adipose tissue, the authors showed that bone marrow-derived macrophages co-cultured with adipose tissue explants from obese mice exhibited enhanced glycolysis and OXPHOS compared with bone marrow derived macrophages exposed to lean adipose tissue^26^. A similar boost of both glycolysis and OXPHOS has been observed in macrophages treated with TLR2 ligands such as Pam3Cys^27^, and this metabolic rewiring in ATM likely results from local cues in the tissue microenvironment. Notably, ATM in the obese adipose tissue are exposed to products of adipocyte lipolysis, such as free fatty acids, and exhibit increased lipid metabolism and lysosomal function^8,14^, which may help fuel fatty acid oxidation^16^. OXPHOS, although slower than glycolysis, also produces greater amounts of ATP from glucose, and has been shown to be required for key macrophage functions such as phagocytosis, which is upregulated as ATM encounter dying adipocytes. Consistent with the coincident upregulation of glycolytic metabolism in ATM, we observed increased levels of its by-product, lactate, in the WAT stromal vascular fraction and serum of obese mice, and high concentrations of lactate have also been reported in obese humans^28^. An upregulation of the glycolytic rate appears to be a common response of activated macrophages challenged with different inflammatory stimuli^12^. Although glycolysis is far less efficient in generating ATP than oxidative phosphorylation, this metabolic adaptation can also feed glycolysis intermediates into the pentose phosphate pathway and provides building blocks needed for macrophage activation, such as amino acids for protein synthesis, ribose for nucleotides, and NADPH for the production of reactive oxygen species^12,15^. Thus, the upregulation of both oxidative phosphorylation and glycolysis programs in ATM likely fuels key functions needed for adaptation of these macrophages in the obese adipose tissue.

Metabolic rewiring of macrophages towards glycolysis has been shown to influence immune cell function. In LPS-stimulated macrophages, accumulation of glycolytic intermediates and ROS can inhibit prolyl hydroxylases that degrade HIF-1α^17^, leading to HIF-1α accumulation and increased expression of the glycolytic machinery. This mechanism of HIF-1α stabilization, termed pseudohypoxia, has been shown to drive expression of IL-1β^17^, a potent inflammatory cytokine that has been implicated in obesity related insulin resistance^11,29^. Notably, we find that ATM isolated from obese mice show a similar pattern of enhanced glycolysis, HIF-1α accumulation and increased IL-1β expression. In the obese adipose tissue microenvironment ATM may be exposed to multiple stimuli that provoke HIF-1α adipose tissue microenvironment ATM may be exposed to multiple stimuli that provoke HIF-1α activation in the absence of reduced oxygen levels. Our data provide indications that free fatty acids released from obese adipocytes and the accumulation of metabolic cycle intermediates, such as succinate and glutamate, could perform such roles. This metabolic program could be provoked *in vitro* by stimulating macrophages with palmitate, a saturated fatty acid that is present at increased levels in the circulation of high-fat-diet-fed mice. Palmitate dose-dependently upregulated the macrophage glycolytic rate, leading to activation of HIF-1α, and increased expression of IL-1β in addition palmitate increased expression of HIF-1α-target genes in the glycolytic pathway, including the glucose transporter *Glut1* and key glycolytic enzymes, which would in turn reinforce aerobic glycolysis. Importantly, we showed that the upregulation of IL-1β and glycolytic genes by palmitate could be blocked by chemical inhibitors of either glycolysis or HIF-1α.

Although our data provide evidence for a role of pseudohypoxia in the induction of IL-1β in ATM, we cannot exclude the contribution of other immune cells, such as eosinophils and dendritic cells, in this context. In this study, we used F4/80 as a marker for macrophages, but this marker can also be expressed by some dendritic cells and eosinophils. Using flow cytometric analysis we showed that 95% of CD11b + F4/80+ cells in obese mice are also positive for the macrophage markers CD64 and MerTK, suggesting that the contribution of other cell types is minor. In addition, HIF-1α activation in ATM is also likely to also be potentiated by low oxygen tensions that occur in the rapidly expanding adipose tissue *in vivo*^20,21^. Indeed, isolation of hypoxic ATM from WAT of obese mice showed high levels of HIF-1α and IL-1β expression, compared to non-hypoxic ATM from the same tissue. Hypoxia is also a driver of glycolysis, and we observed increased expression of the glucose transporter *Glut1* and glycolytic enzymes in hypoxic compared to non-hypoxic ATM. These data suggest that multiple mechanisms likely sustain glycolysis in ATM, with activation of HIF-1α and induction of IL-1β expression as a common consequence of this metabolic reprogramming.

Considerable evidence points to a role for IL-1β in the development of insulin resistance and type 2 diabetes. Low grade inflammation has been shown to precede the development of type 2 diabetes, and elevated levels of IL-1β in the circulation are associated with increased risk^30^. Studies using obesity- and diabetes-associated ligands have identified roles for palmitate^11^, insulin associated polypeptide (IAPP)^31,32^, and ceramide^29^ in activating the NLRP3 inflammasome complex required for IL-1β maturation and secretion. IL-1β is a potent inflammatory cytokine that can amplify the production of other inflammatory mediators, such as TNF-a and IL-6, via signalling through the IL-1 receptor, and can also increase hematopoiesis^33^. Furthermore, studies that block IL-1β production using genetic deletion of components of the NLRP3 inflammasome that regulates IL-1β maturation or inhibition of the IL-1β signaling machinery have established a role for this cytokine in promoting insulin resistance and metabolic dysfunction in high fat diet fed mice^11,29,34^, as well as conditions accelerated by diabetes such as atherosclerosis^32,35,36^. These studies have led to thinking that pharmacological approaches to treat diabetes should not only correct hyperglycaemia, but also attenuate inflammation to prevent the development of metabolic and cardiovascular complications.

To test whether targeting macrophage immunometabolism could block the production of IL-1β and its inflammatory and metabolic sequelae, we generated mice lacking HIF-1α specifically in myeloid cells and challenged them to high diet feeding. We found that targeted deletion of myeloid HIF-1α significantly reduced macrophage accumulation in the WAT, without altering weight gain or adiposity, as compared to WT mice. Furthermore, mice with myeloid HIF-1α deficiency showed lower levels of IL-1β expression in both WAT and the circulation than WT mice, consistent with HIF-1α being a key mechanism underlying IL-1β expression in the setting of obesity. The reduction of systemic levels of IL-1β in myeloid HIF-1α KO mice implicates macrophages as a major source of this cytokine *in vivo* during high fat diet feeding. Moreover, the reduced ATM accumulation in myeloid HIF-1α null mice suggests that the HIF-α–IL-1β axis amplifies macrophage burden in the WAT, a finding supported by previous studies showing that IL-1β promotes myelopoiesis, thereby enhancing the supply of monocytes to WAT^33^. In addition to the decrease in IL-1β expression in ATM, we observed an increase in markers of alternatively activated M2 macrophages in the WAT of HFD-fed mice with myeloid deficiency of HIF-1α compared to WT mice, suggesting that loss of HIF-1α leads to a less inflammatory macrophage phenotype in the adipose tissue. As HIF-1α drives glycolysis, this shift in ATM polarization toward an M2-like macrophage phenotype in its absence may result from a metabolic reprogramming toward OXPHOS, which has been shown to drive alternative activation^16,18^.

Despite reducing WAT inflammation and systemic levels of IL-1β, the conditional deletion of HIF-1α in myeloid cells, however, was not sufficient to reverse the impairment of glucose clearance and insulin tolerance associated with high fat diet feeding in this study. Notably, our findings differ from a recent report that myeloid deficiency of HIF-1α improved systemic insulin resistance, as well as WAT inflammation, in a similar model of diet-induced obesity^37^. While the reasons for these discrepant findings are unclear, they may arise from differences in the diet used or the microbiome of the mice housed in different facilities. Nevertheless, studies of selective HIF-1α-deficiency in other cell types in the adipose tissue, most notably adipocytes, has been shown to protect against insulin resistance^38^, suggesting that targeting HIF-1α broadly in WAT may be a strategy to improve both inflammation and metabolic dysfunction. Collectively, our data identify enhanced glycolysis and HIF-1α activation in ATM as key mechanisms underlying macrophage persistence in the WAT and the local and systemic expression of IL-1β, placing these immunometabolic signals at the interface of macrophage activation in obesity.

## Materials and Methods

### Mice

The Institutional Animal Care and Use Committee of New York University Medical Center approved all animal experiments. All methods were performed in accordance with New York University Medical Center guidelines and regulations. The Hif1α^fl/fl^ and LysMCre transgenic mice were purchased from Jackson laboratory and mated to generate Hif1α^fl/fl^LysMCre+ mice (HKO) and Hif1α^fl/fl^LysMCre− control mice. The mice were maintained under a standard light cycle (12 h light/dark) and were allowed free access to water and food. They were fed a low fat chow diet 13.2% calories from fat, 24.6% protein, 62.1% carbohydrate (PicoLab Rodent Diet 20) or a high fat diet (HFD) containing 60% calories from fat, 20% protein, 20% carbohydrate (Research Diets, D12492). After feeding, the body composition and % adiposity of mice was analysed by dual-energy X-ray absorptiometry (DEXA) scanning. Glucose-tolerance tests were performed as previously described^23^. After determination of fasted blood glucose levels, each animal received a glucose gavage of 1.5 g/kg body weight of glucose (25% D-glucose, G7528; Sigma Aldrich). Blood glucose levels were determined after 15, 30, 60 and 120 min. Insulin-tolerance tests were carried on unfasted animals by injecting an i.p. injection of 1.5 U/kg body weight of insulin (HumilinR 100 U/ml). Blood glucose levels were detected after 15, 30, 60, 120 and 240 mins. Interleukin (IL)-1β and IL-6 levels in the serum were measured by cytometric bead array (CBA) (BD biosciences, NJ, USA) as we described^39^, and lactate levels in the serum and stromal vascular cell fractions by ELISA kit (Lactate Colorimetric/Fluorometric Assay Kit, BioVision Inc.) respectively according to the manufacturer’s guidelines.

### Hypoxia probe

Hypoxyprobe^TM^ (pimonidazole hydrochloride; Hypoxyprobe, Inc. MA, USA) immunohistochemical and flow cytometry analyses were used to assess hypoxia in cells and tissues as we previously described^25^. Pimonidazole is a 2-nitroimidazole that is reductively activated in hypoxic cells and forms stable adducts with thiol groups in proteins, peptides, and amino acids^40^. Pimonidazole hydrochloride was injected intraperitoneally at a dose of 120 mg/kg body weight 60 min before tissue collection. Anti-pimonidazole antibody (clone 4.3.11.3, MAb1, Hypoxyprobe, Inc. MA, USA) was used to detect pimonidazole positive regions.

### Primary cell culture

For adipose tissue macrophage purification, visceral fat pads of mice fed were excised, minced in Hank’s Balanced Salt Solution (HBSS) and centrifuged at 500 g for 5 minutes to remove erythrocytes and free leukocytes. Liberase (5401119001; Roche applied science) was added to tissue suspensions at 1 mg/ml and incubated at 37 °C for 30 minutes with shaking. The tissue suspension was filtered through a 100-μm filter and then spun at 300 g for 5 minutes to separate floating adipocytes from the stromal vascular fraction (SVF) pellet. Isolation of CD11b+ cells from the SVF was performed by magnetic immunoaffinity isolation with anti-CD11b antibodies conjugated to magnetic beads (10 µl/10^7^ cells, 120-000-300; MACS Miltenyi Biotec). Cells were isolated using positive selection columns (LS Columns, 130-0420401, MACS Miltenyi Biotec) prior to preparation of whole-cell lysates.

For primary bone marrow derived macrophages (BMDM) preparation, the bone marrow of the tibia and femur of 6–8 week old C57Bl/6 mice was flushed using ice-cold DMEM supplemented with 10% FBS and 1% penicillin/streptomycin. Cells were plated overnight in DMEM supplemented with 20% L929-conditioned media, 10% FBS and 1% penicillin/streptomycin, and non-adherent cells were removed and cultured further for 7 days as we described^18^. For Seahorse extracellular flux (XF) analysis, BMDM were stimulated with 50–250 µM BSA-conjugated palmitate or BSA as a control for 24 h and the Seahorse XF Glycolysis Stress Test (ECAR; extracellular acidification rate) and Seahorse XF Mito stress test (OCR; oxygen consumption rate) (Agilent technologies, CA, USA) were performed as described previously^18^. For gene expression analyses, BMDM were treated with 250 µM BSA-conjugated palmitate or BSA for 24 hours and RNA was isolated. In some assays, BMDM were also treated with 2-deoxyglucose or HIF-1α inhibitor for 24 hours (100 µM, #400083, Calbiochem).

### Palmitate-BSA preparation

Sodium palmitate (P9767; Sigma Aldrich, MO, USA) was dissolved in sterile water to make a 100 mM solution by alternating heating and vortexing until the palmitate was dissolved: this occurred once the solution reached 70 °C. Immediately after the palmitate dissolved, it was conjugated to serum-free DMEM containing 5% NEFA-free BSA, creating a 5 mM palmitate solution as described^11,23^. The 5 mM palmitate solution was shaken at 140 rpm at 40 °C for 1 h and was then immediately used to treat the cells. Serum-free DMEM containing 5% NEFA-free BSA was used as the vehicle control.

### Real-time quantitative RT-PCR analysis

Total RNA was isolated using TRIzol reagent (Invitrogen) and Direct-zol RNA MiniPrep columns (Zymo Research). For mRNA quantification, RNA (0.5–1 µg) was reverse transcribed using iScript^TM^ cDNA Synthesis Kit (Biorad, CA, USA) and quantitative real-time (qRT) PCR was performed in triplicate using kappa SYBR green Supermix (Kappa Biosystems, MA, USA) and a QuantStudio 3 Real-Time PCR system (ThermoFisher Scientific, MA, USA) as we previously described^23^. The primers used are listed in Supplemental Table 1. Fold change in mRNA expression was calculated normalized to 28S using the comparative cycle method (ΔΔCt).

### Immunohistochemistry

White adipose tissue was excised, fixed in formalin overnight, embedded in paraffin and sectioned. The immunofluorescence analysis of F4/80 (MCA497GA; Abd Serotec, NC, USA), lactate dehydrogenase (ab47010, Abcam)  and HIF-1α (NB100-479; Novus Biologicals, CO, USA) was conducted after deparaffinization as described previously^23^. Sections were mounted and visualized using a Nikon Eclipse microscope.

### Western-blot analysis

BMDMs treated with palmitate were homogenized in lysis buffer as previously described and subjected to SDS-PAGE analysis^23,25^. Western blot analysis was carried out using anti-HIF-1α (Novus Biologicals) and α-tubulin (T6074; Sigma Aldrich) as loading control.

### Statistical methods

The difference between two groups was analyzed by Student’s t-test or for multiple comparisons, by one-way analysis of variance, followed by Newman-Keus multiple comparison test. P values of less than 0.05 were considered significant.

## Supplementary information

Supplementary data.

Supplementary data2.
