# Supplementary material for: Enhanced glycolysis and HIF-1α activation in adipose tissue macrophages sustains local and systemic interleukin-1β production in obesity
Source: Sci Rep. 2020 Mar 27;10:5555. doi: 10.1038/s41598-020-62272-9 (PMC7101445; doi:10.1038/s41598-020-62272-9)
Supplement: Supplementary file 1 — Supplementary data. [file 41598_2020_62272_MOESM1_ESM.pdf]

Supplementary Data File

Enhanced glycolysis and HIF-1α activation in adipose tissue macrophages sustains local and systemic interleukin-1β production in obesity

Monika Sharma[1], Ludovic Boytard[2], Tarik Hadi[2], Graeme Koelwyn[1], Russell Simon[1], Mireille Ouimet[1], Lena Seifert[2], Westley Spiro[1], Bo Yan[1], Susan Hutchison[1], Edward A. Fisher[1], Ravichandran Ramasamy[3], , Bhama Ramkhelawon[2*], Kathryn J Moore[1*]

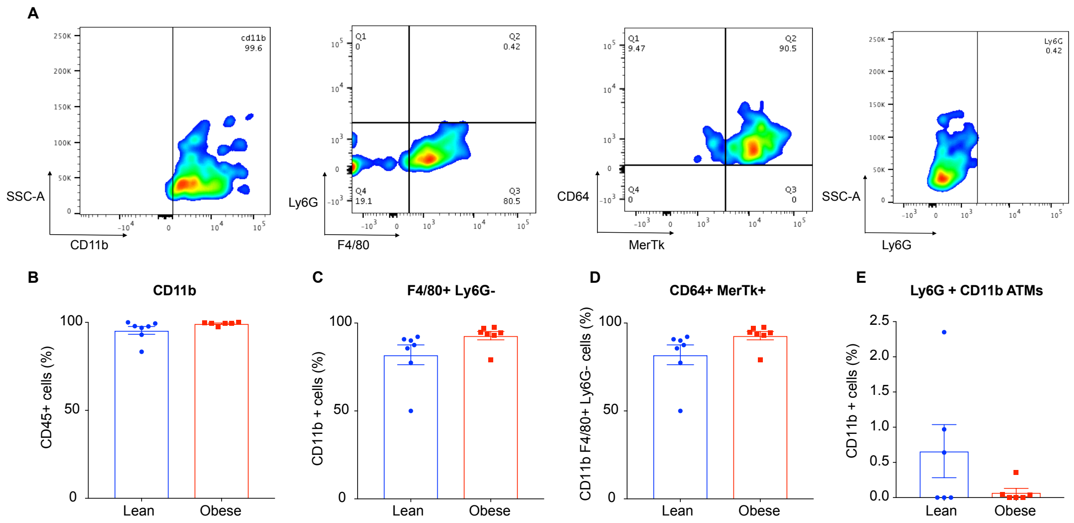

Supplementary Figure 1: A) Gating strategy for flow cytometeric analysis of CD11b, F4/80, Ly6G, CD64, MerTk populations performed using Flowjo software. Flow cytometric quantification of **B)** CD11b, **C)** F4/80+Ly6G-, **D)** CD64+MerTk+, and **E)** Ly6G in ATMs from lean and obese wild type (WT) mice (n=6-7).

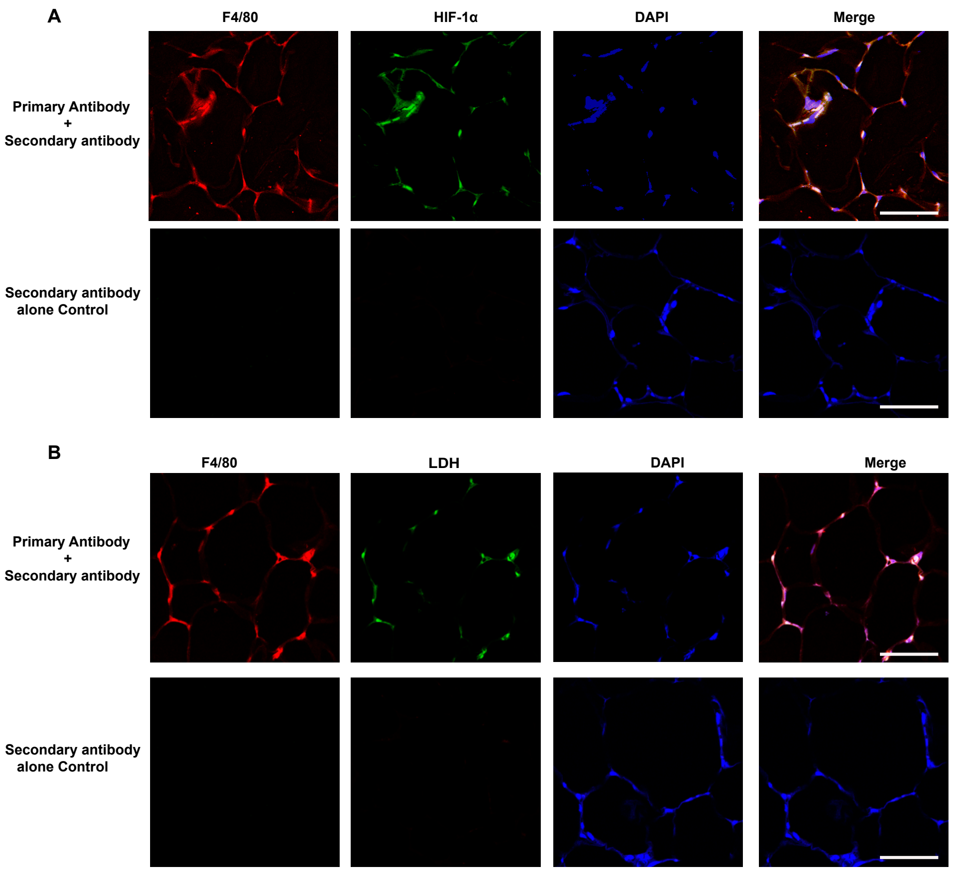

Supplementary Figure 2: Representative immunofluorescence staining of the **A)** macrophage marker F4/80 (red), HIF-1α (green), DAPI (blue), and **B)** macrophage marker F4/80 (red), lactate dehydrogenase (LDH; green), DAPI (blue) in WAT of wild type mice fed HFD for 20 weeks. Colocalization of HIF-1α with F4/80 and DAPI is shown in the merged image (arrows). Lower panel shows secondary antibody alone controls. Scale bar =100 µm.

Supplementary Figure 3

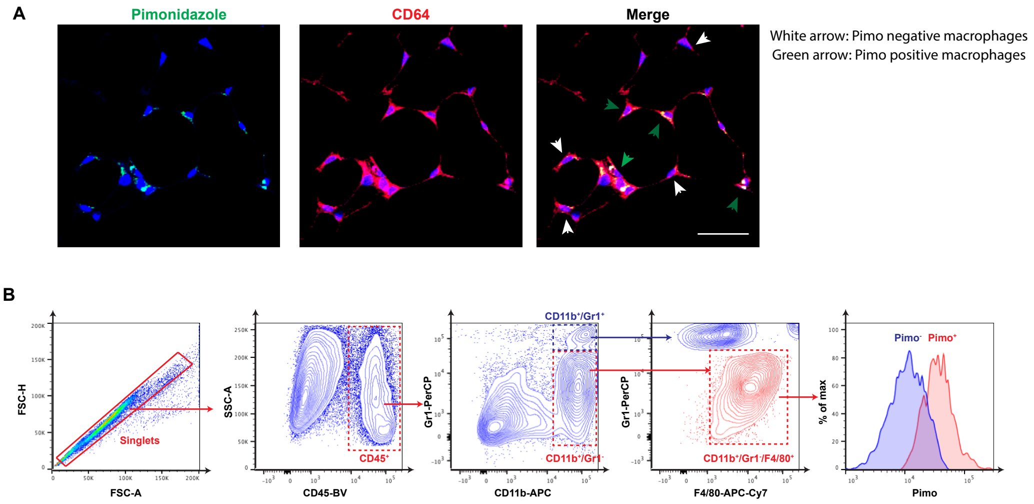

Supplementary Figure 3: A) Representative images of immunofluorescence staining for pimonidazole (green), CD64 (red) and DAPI nuclear stain (blue) in WAT of obese wild type mice. Scale bar = 100 µm. **B)** Gating strategy for flow cytometer as shown for singlets, CD45, CD11b, F4/80 and pimonidazole populations performed by Flowjo analysis.

Supplementary Figure 4

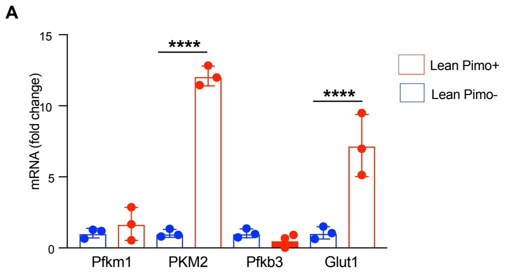

Supplementary Figure 4: Relative mRNA expression of *Pfkm1*, *Pkm2*, *Pfkb3* and *Glut1* in Pimonidazole (Pimo)– and Pimo+ ATMs isolated from lean mice (n=3). Data are mean ± s.e.m. ****p < 0.001.

Supplementary Figure 5

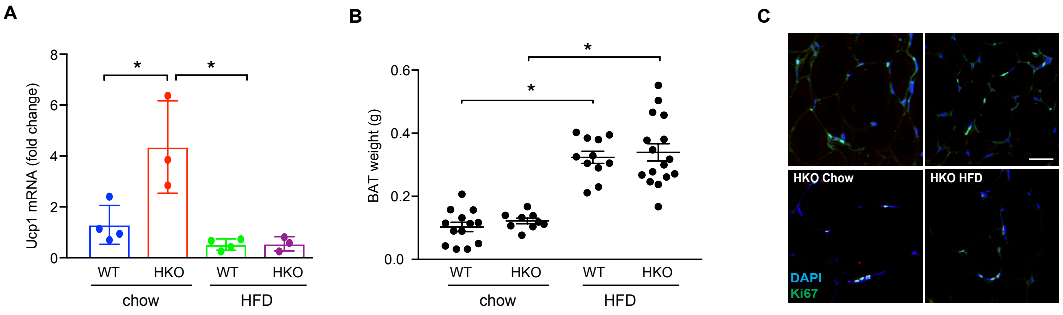

Supplementary Figure 5: A) Fold change in *Ucp1* mRNA in WAT of lean and obese WT and HKO mice. **B)** BAT weight of WT and HKO mice after 20 weeks of chow and HFD diet. **C)** Immunofluorescence staining of Ki67 (green) and DAPI nuclear stain (blue) in WAT of lean and obese WT and HKO mice. Scale bar = 100 µm.
