# Supplementary material for: Enhanced glycolysis and HIF-1α activation in adipose tissue macrophages sustains local and systemic interleukin-1β production in obesity
Source: Sci Rep. 2020 Mar 27;10:5555. doi: 10.1038/s41598-020-62272-9 (PMC7101445; doi:10.1038/s41598-020-62272-9)
Supplement: Supplementary file 2 — Supplementary data2. [file 41598_2020_62272_MOESM2_ESM.docx]

**Enhanced glycolysis and HIF-1**α **activation in adipose tissue macrophages sustains local and systemic interleukin-1β production in obesity**

Monika Sharma^1^, Ludovic Boytard^2^, Tarik Hadi^2^, Graeme Koelwyn^1^, Russell Simon^1^, Mireille Ouimet^1^, Lena Seifert^2^, Westley Spiro^1^, Bo Yan^1^, Susan Hutchison^1^, Edward A. Fisher^1^, Ravichandran Ramasamy^3^, Bhama Ramkhelawon^2*^, Kathryn J Moore^1*^

Supplemental Table1.

| Primer | Forward | Reverse |
| --- | --- | --- |
| *Hif1a* | ACCTTCATCGGAAACTCCAAAG | CTGTTAGGCTGGGAAAAGTTAGG |
| *Hif2a* | CTGAGGAAGGAGAAATCCCGT | TGTGTCCGAAGGAAGCTGATG |
| *Vegfα* | CCACGACAGAAGGAGAGCAGAAGTCC | CGTTACAGCAGCCTGCACAGCG |
| *Glut1* | CAGTTCGGCTATAACACTGGTG | GCCCCCGACAGAGAAGATG |
| *Fizz1* | CCTGCTGGGATGACTGCTA | TGGGTTCTCCACCTCTTCAT |
| *Arg1* | GGTCCACCCTGACCTATGTGT | ACGATGTCTTTGGCAGATATGC |
| *Cd206* | CTCTGTTCAGCTATTGGACGC | CGGAATTTCTGGGATTCAGCTTC |
| *Il1β* | TGTGAATGCCACCTTTTGACA | GGTCAAAGGTTTGGAAGCAG |
| *Il6* | CCAAGAGGTGAGTGCTTCCC | CTGTTGTTCAGACTCTCTCCCT |
| *Tnfα* | TTAAAAACCTGGATCGGAACCAA | GCATTAGCTTCAGATTTACGGGT |
| *Ccl2* | ATGAGCACAGAAAGCATGATCCGC | CCAAAGTAGACCTGCCCGGACTC |
| *Pfkm1* | TGTGGTCCGAGTTGGTATCTT | GCACTTCCAATCACTGTGCC |
| *Pkm2* | AGGATGCCGTGCTGAATG | TAGAAGAGGGGCTCCAGAGG |
| *Pfkb3* | CCCAGAGCCGGGTACAGAA | GGGGAGTTGGTCAGCTTCG |
| *28s* | TGGGGAATGCAGCCCAAG | CCTTACGGTACTTGTTGACTATGC |

Table 1: List of primer sequences used for quantitative PCR reactions.
